# Antioxidant and Emulsifying Activity of the Exopolymer Produced by *Bacillus licheniformis*

**DOI:** 10.3390/ijms25158249

**Published:** 2024-07-28

**Authors:** Sánchez-León Enrique, Amils Ricardo, Abrusci Concepción

**Affiliations:** 1 Departamento de Biología Molecular, Facultad de Ciencias, Universidad Autónoma de Madrid, UAM, Cantoblanco, 28049 Madrid, Spain; 2 Centro de Biología Molecular Severo Ochoa, CSIC-UAM, 28049 Madrid, Spain

**Keywords:** antioxidants, emulsifying, exopolymer, negative charges, toxicity

## Abstract

The exopolymer (ESPp) was obtained from *Bacillus licheniformis* IDN-EC, composed of a polyglutamic acid and polyglycerol phosphate chain O-substituted with αGal moieties (αGal/αGlcNH_2_ 3:1 molar ratio) and with a 5000 Da molecular weight. The cytotoxicity activity of EPSp was determined by reducing the MTT (3-[4,5-dimethyl-thiazol-2-yl]-2,5-diphenyltetrazolium bromide) to formazan on HeLa cells. This EPS did not show cytotoxicity against the tested cell line. The ESPp presented great advantages as an antioxidant with free radical scavenging activities (1,1-diphenyl-2-picryl-hydrazyl radical (DPPH),hydroxyl radical (OH), and superoxide anion (O_2−_)) (65 ± 1.2%, 98.7 ± 1.9%, and 97 ± 1.7%), respectively. Moreover, EPSp increased the enzyme activity for catalase (CAT) and glutathione peroxidase (GSH-Px) in HeLa cells (CAT, 2.6 ± 0.24 U/mL; and GSH-Px, 0.75 ± 0.3 U/L). The presence of ESPp showed a significant protective effect against H_2_O_2_ in the cell line studied, showing great viability (91.8 ± 2.8, 89.9 ± 2.9, and 93.5 ± 3.6%). The EPSp presented good emulsifying activity, only for vegetable oils, olive oil (50 ± 2.1%) and sesame (72 ± 3%). Sesame was effective compared to commercials products, Triton X-100 (52.38 ± 1.6%), Tween 20 (14.29 ± 1.1%), and sodium dodecyl sulphate (SDS) (52.63 ± 1.6%). Furthermore, the EPS produced at 0.6 M has potential for environmental applications, such as the removal of hazardous materials by emulsification whilst resulting in positive health effects such as antioxidant activity and non-toxicity. EPSp is presented as a good exopolysaccharide for various applications.

## 1. Introduction

The search for bacteria capable of producing exopolymers with biotechnological applications has focused in recent years on different environments [1,2,3]. The existence of hundreds of millions of small microbial factories, capable of synthesizing polymers with a high biotechnological potential, is enormously attractive. One of the most important aspects to consider in the search for biotechnological applications is the knowledge of the main components of the polymer. The composition of the polymer allows us to understand the possible mechanisms of action and, therefore, the most efficient applications at an industrial level. The polymers synthesized by bacteria are mainly exopolysaccharides (EPS). Cataloguing exopolysaccharides is complex, and their classification must consider their chemical nature.

**Figure 1:**
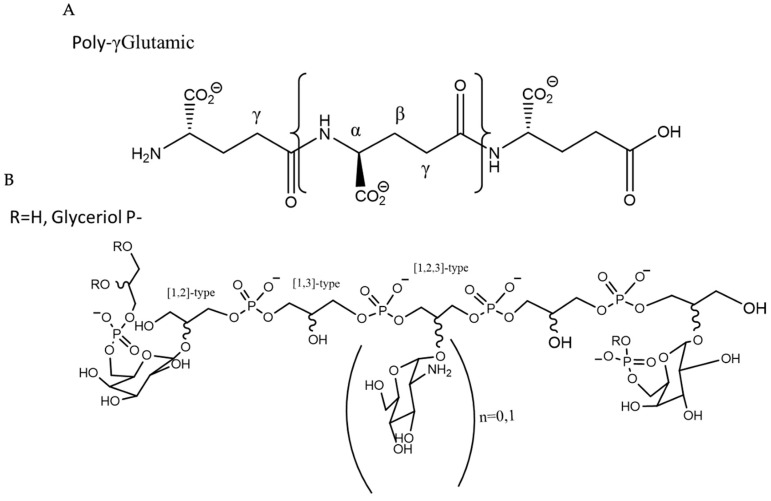
Structure of EPSp: (**A**) polyglutamic acid and (**B**) polyglycerol phosphate chain O-substituted with αGal moieties (αGal/αGlcNH2 3:1 molar ratio).

Homopolysaccharides are composed of a single type of monosaccharide (α-d-glucans and β-d-glucans) and heteropolysaccharides are composed of more than one different type of monomer, such as fructans and polygalactan [4,5]. Polysaccharides can be composed of both organic and inorganic molecules. Common organic compounds are acetates, pyruvates, and amino acids, whilst the most widespread inorganic compounds are sulphates and phosphates. The advantages offered by bacterial exopolysaccharides make them an interesting alternative to synthetic compounds, such as a less complex purification compared to vegetable polymers, their low cost, their biodegradability, and their lack of toxicity [6]. They have potential in a wide spectrum of industrial and biotechnological applications, both from the environmental point of view, as emulsifiers, polyanionics, gelling agents, and pseudoplastics, and from the pharmaceutical and food industries’ perspective, as antioxidants, antimicrobials, and anticancer treatments [7,8,9,10,11]. However, the exploration of these exopolysaccharides produced by bacteria is focused on very specific niches, which can hinder a broader knowledge of their possible applications [12]. The search for micro-organisms with a wide spectrum in industrial applications opens up an interesting avenue for its exploration. Within the phylum Bacillota, the aerobic micro-organisms of the genus *Bacillus* are considered to be of interest due to its high level of diversity [13], and *Bacillus licheniformis*, being a well-characterized species of this genus [14]. This micro-organism is capable of colonizing very diverse environments, due to its wide range of adaptative parameters (temperature, oxygen, pH, ionic strength, etc.). In this sense, it is not surprising that this species can be a common organism in very diverse and disparate habitats such as soils, hot springs, plants, geothermal sediments, sea, and other very diverse sources [15]. For all these reasons, *Bacillus licheniformis* is a very attractive species to be tested for its use in different industrial processes [16,17]. In addition, the possible effect that the negative charges of the exopolymers can have in their different applications is an interesting hypothesis to test. The aim of this work was to evaluate the biotechnological possibilities of the purified exopolymer, EPSp, produced by *B. licheniformis* IDN-EC that contained polyglutamic acid and an acidic polysaccharide in its composition. This was formed by an O-substituted polyglycerol phosphate chain with αGal residues in terminal positions and further modified with αGlcNH_2_ with a molar ratio of 3:1, which were both negatively charged (Figure 1) [18]. For this, a series of investigations was carried out to determine the influence of this composition in cytotoxicity, antioxidant, and bioremediation applications.

## 2. Results and Discussion

The aim of this work was to investigate the biotechnological potential of the purified exopolymer, EPSp, produced by *B. licheniformis* IDN-EC. The ESPp was obtained from the procedure indicated in Section 3.2. The chemical composition of the EPSp was determined as described in a previous work [18].

### 2.1. Cytotoxicity

**Figure 2:**
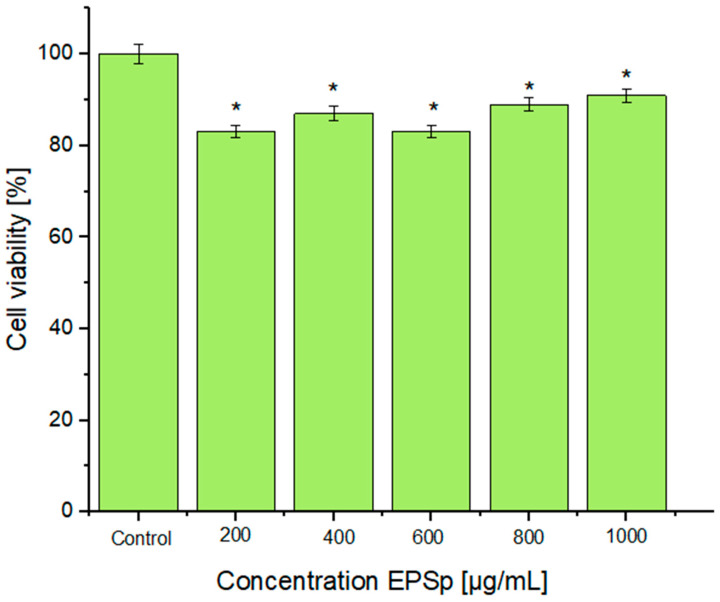
Biocompatibility (MTT) of EPSp with HeLa cell. * Indicating significant differences between control and concentration of EPSp (*p* < 0.05).

The cytotoxicity of EPSp in HeLa cells treated with the different concentrations of EPSp (200, 400, 600, 800, and 1000 μg/mL) is shown in Figure 2. The cell viability was above 80% very close to the control, and in no case was the cell proliferation compromised. These results demonstrated that the negative charges of the EPSp were not detrimental. The EPSp was less cytotoxic when compared to those obtained by other strains of the same species. This was the case for EPS-1 from *B. licheniformis* B3-15 with cytotoxicities of 29% at 500 μg/mL [19], whose EPS-1 was composed mainly of mannose, and from *B. licheniformis* PASS26, with a cytotoxicity of 46.2% at 800 μg/mL [20], composed of mainly mannose and galacturonic acid.

### 2.2. Free Radical Scavenging Activities

**Figure 3:**
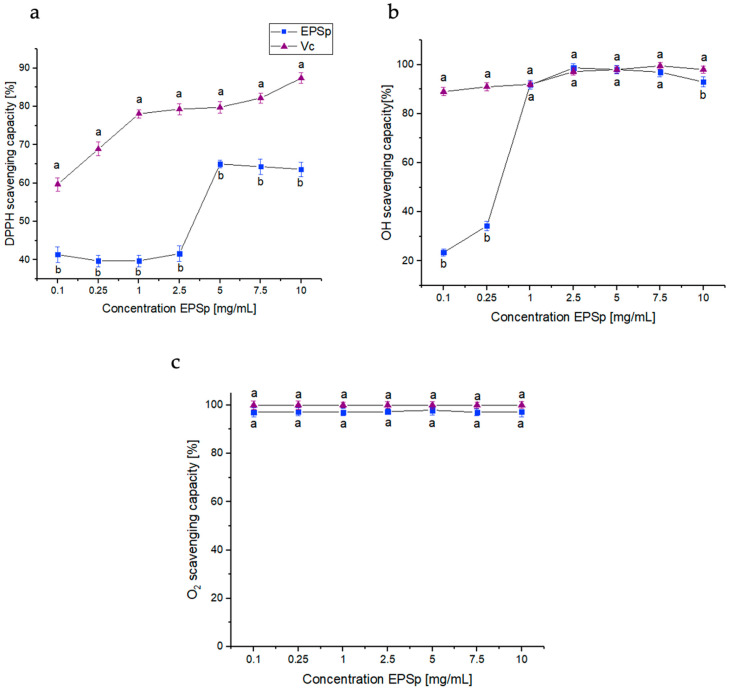
Non-enzymatic antioxidant radical scavenging activity: (**a**) 1,1-diphenyl-2-picryl hydrazyl radical (DPPH), (**b**) hydroxyl radical (OH), and (**c**) superoxide radicals (O_2−_). Different letters (**a**,**b**) indicate significant differences between concentration of Vc and EPSp (*p* < 0.05).

The EPSp free radical scavenging activities, 1,1-diphenyl-2-picryl hydrazyl radical (DPPH), hydroxyl radical (OH), and superoxide radicals (O_2−_), is shown in Figure 3. EPSp was analyzed in a range of 0.1 to 10 mg/mL, using ascorbic acid (Vc) as a positive control. The free radical scavenging activity of DPPH is shown in Figure 3a. The best activity was obtained between 5 and 10 mg/mL with a radical scavenging of 65 ± 1.2% and 63.6 ± 1.9%, respectively. This activity was higher than that presented by other *B. licheniformis* strains with an activity for its EPS20 of 35% at 10 mg/mL [21]. In the case of the OH scavenging activity (Figure 3b), the activity remained stable with a value of 90% in all concentrations, except at 2.5 mg/mL, which reached its maximum activity with 98.7 ± 1.7%. This activity was superior to that shown in *B. licheniformis* OSTK95 (50.9%), *Bacillus velezensis* SN-1 (58.9%), and *Bacillus haynesii* CamB6 (76.21%) [22,23,24]. In addition, ESPp showed excellent activity against O_2−_ (Figure 3c), with 97% in all concentrations tested. The activity presented by EPSp was superior to other exopolymers of *B. licheniformis* OSTK95 (43.89%) [24] and *B. licheniformis* KS-20 (28%) [21]. The difference between the three antioxidant activities (DPPH; OH; and O_2−_) could be due to the chemical composition of the functional groups of EPSp. The availability to transfer electrons and to donate protons, or the physical structure of the EPSp molecule could contribute to these differences between antioxidant activities [25].

### 2.3. H_2_O_2_-Induced Assay, and Effects of the Exopolymer

**Figure 4:**
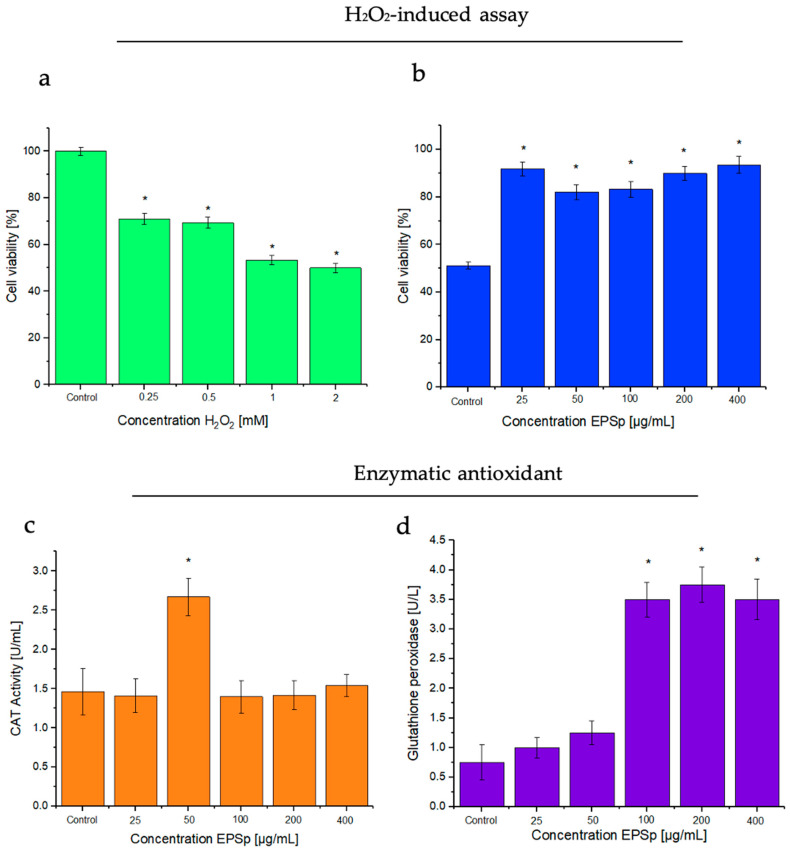
H_2_O_2_-induced assay: (**a**) evaluation of the H_2_O_2_-damaged HeLa cells, and (**b**) evaluation of the protection of H_2_O_2_-damaged HeLa cells protection with EPSp. Enzymatic antioxidant activity: (**c**) catalase activity in the presence of EPSp, and (**d**) glutathione peroxidase activity in the presence of EPSp. * Indicates significant differences between control and concentration of EPSp (*p* < 0.05).

The effects of the exopolymer against H_2_O_2_, and enzymatic antioxidant assays in HeLa cells are shown in Figure 4. The assay induced by H_2_O_2_ (Figure 4a) in HeLa cells had marked oxidative stress; at the 2 mM concentration, cell viability was reduced to 50%. From this reduction, the effects of the different concentrations of ESPp against H_2_O_2_ were tested (Figure 4b). The results indicated a significant protective effect of the EPSp at concentrations of 25, 200, and 400 μg/mL, showing a viability of 91.8 ± 2.8, 89.9 ± 2.9, and 93.5 ± 3.6%, respectively, with significant differences in relation to the control. Similar results were shown by the exopolysaccharide LPC-1 from *Lactobacillus plantarum* C88, in Caco-2 cells, showing protection with concentrations between 50 and 200 μg/mL, where a significant reduction in reactive oxygen species caused by H_2_O_2_ was observed [26]. This reduction in injuries could be due to the presence of negative charges in the exopolymer, which allowed for a significant increase in cell protection.

### 2.4. Enzymatic Antioxidant Assays: Catalase (CAT) and Glutathione Peroxidase (GSH-Px)

Assays for the enzymatic antioxidants CAT and GSH-Px are shown in Figure 4c,d. The presence of EPSp increased the enzyme activity in HeLa cells. In the case of CAT activity, the results showed a significant activity of 2.6 ± 0.24 U/mL in 50 μg/mL of EPSp, higher than that presented in the control (1.48 ± 0.3 U/mL). In the case of GSH-Px, the results showed an activity of 3.5 ± 0.29, 3.7 ± 0.3, and 3.5 ± 0.34 U/L in the 100, 200, and 400 μg/mL concentrations, respectively, of EPSp. These were significantly higher compared to control (0.75 ± 0.3 U/L). These results are more favorable than those presented by other species of the same genus such as *Bacillus cereus* SZ1 with a CAT activity of 1.5 U/mg at 300 μg EPS, compared to its control (0.9 U/mg) [27]. These results could demonstrate that the negative charges of the EPSp favored the increase in the enzymatic activity of CAT and GSH-Px with respect to their control. The increase in the antioxidant activity of EPSp makes it a candidate to cancel the toxic activity caused by hydrogen peroxide, maintaining the oxidative homeostasis of cells [28,29].

### 2.5. The Emulsifying Capacity of EPSp

**Figure 5:**
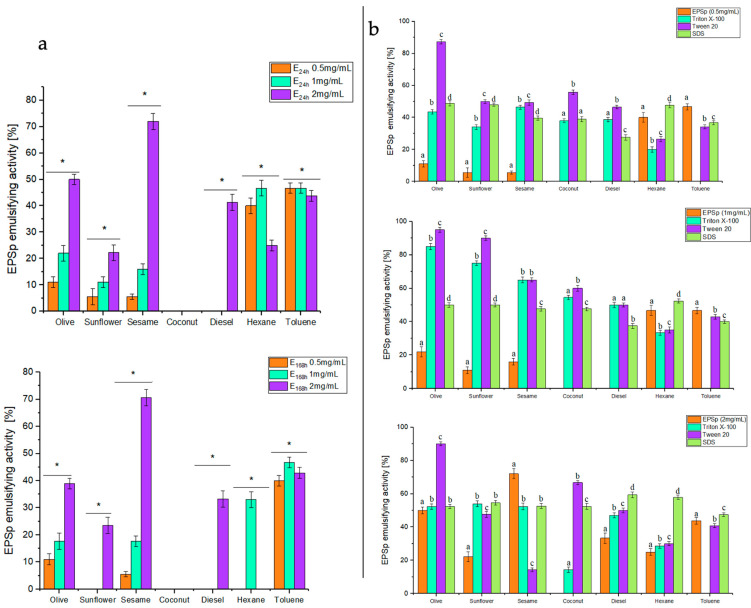
Emulsifying activity of EPSp at concentrations of 0.5, 1, and 2 mg/mL: (**a**) emulsifying activity with the different natural oils and hydrocarbons used at 24 h (E24) and 168 h (E168) (* *p* < 0.05), (**b**) comparison of emulsifying activity at different concentrations of EPSp versus commercial emulsifiers (Triton X-100, Tween 20, and SDS) across different natural oils and hydrocarbons at 24 h (E24). Different letters (**a**–**d**) represent the statistical difference between different emulsifiers for each natural oil and hydrocarbon (*p* < 0.05).

The emulsifying capacity of EPSp (Figure 5) was evaluated to verify the bioremediation capacity of the exopolymer. The emulsifying capacity was tested with common natural oils (sunflower, olive, sesame, and coconut) and hydrocarbons (diesel, hexane, and toluene) used in the industry. In addition to this, a comparison was made between EPSp and commonly used commercial emulsifiers (Triton X-100, Tween 20, and SDS), at two different times (24 and 168 h), in different concentrations (0.5, 1, and 2 mg/mL), and at pH 7.0. Figure 5a shows that EPSp was not effective for natural oils and hydrocarbons at concentrations of 0.5 and 1 mg/mL, for any of the times tested. In the case of the 2 mg/mL concentration, the emulsifying activity of the EPS was significantly (*p* < 0.05) improved. The EPSp presented good emulsifying activity, equal to or greater than 50%, only for natural olive (50 ± 2.1%) and sesame (72 ± 3%) oils at 24 h, extending this emulsifying activity for sesame (70.6 ± 3.2%) to 168 h. On the other hand, EPSp did not show significant emulsifying activity compared to the commercial surfactants tested (Figure 5b). Only at a concentration of 2 mg/mL at 24 h did it present good activity, again with olive oil (50 ± 2.1%) and sesame (72 ± 3.2%). In addition, in the case of sesame (72 ± 3.2%), it could be considered very effective compared to the commercial products Triton X-100 (52.38 ± 1.6%), Tween 20 (14.29 ± 1.1%), and SDS (52.63 ± 1.6%). Similar results were found in the EPS1 of *B. licheniformis* MS3, for peanut oil (54%) [30]. However, in the *B. licheniformis* PASS26 strain, its EPS had emulsifying capacities for a greater number of natural oils (olive, sunflower, peanut, soybean, coconut, mustard, and rice) (70%) and for hydrocarbons (kerosene and hexane) (60%) [20]. These differences may be associated with the chemical composition of the polymer since the presence of negative charges in the EPSp could significantly affect the emulsifying capacity of the polymer [20,30,31,32].

## 3. Materials and Methods

### 3.1. Materials

The expolymer ESPp was obtained from *Bacillus licheniformis* IDN-EC (Accession #HM055601) [33]. The composition of the exopolymer was obtained following our previously described method [18].

Glucose, trypticase soy agar (TSA), DEAE-52 anion, 1,1-diphenyl-2-picryl hydrazyl radical (DPPH), hydrogen peroxide (H_2_O_2_), salicylic acid, pyrogallol, hydrochloric acid (HCl), potassium hydroxide (KOH), ferrous sulphate (FeSO_4_), phosphate-buffered saline (PBS), vitaminic C (Vc), catalase kit, gluthatione peroxidase, MTT (3-[4,5-dimethyl-thiazol-2-yl]-2,5-diphenyltetrazolium bromide) kit, vegetable oils, hydrocarbons, polyoxyethylene sorbitan monolaurate (Tween 20), 2-[4-(2,4,4-trimethylpentan-2-yl)phenoxy]ethanol (Triton X-100), and sodium dodecyl sulphate (SDS) were from Sigma-Aldrich, Madrid, Spain. The HeLa cells (human T-cell lymphoblast-like cell line) were obtained from the Centro de Biología Molecular Severo Ochoa (CBM-UAM), Universidad Autónoma de Madrid, Madrid, Spain. Dulbecco’s modified Eagle medium (H-DMEM), fetal bovine serum (FBS), L-glutamine, penicillin, and streptomycin were from Aldrich, Schnelldorf, Germany.

### 3.2. Production, Isolation, and Purification of the Exopolymer

The production, isolation, and purification of the exopolymer were carried out following our previously described method [18]. The strain was grown in a trypticase soy agar (TSA) medium and incubated at 45 °C for 24 h and moved into flasks of 100 mL filled with 20 mL of a minimal growth medium (MGM) [34]: g/L: K_2_HPO_4_ 0.5, KH_2_PO_4_ 0.04, NaCl 0.1, CaCl_2_ 2H_2_O 0.002, (NH_4_) 2SO_4_ 0.2, MgSO_4_ 7H_2_O 0.02, and FeSO_4_ 0.001, with glucose (4 g/L), and pH adjusted to 7.0. The flasks were incubated at 45 °C and 110 rpm for 24 h. Subsequently, and under the same conditions, 10 mL of this broth (concentration of 2.5 × 10^7^ cells/mL) were inoculated into flasks containing 1000 mL of MGM and glucose. Three independent trials were performed.

The cultures were centrifuged at 13,154× *g* for 30 min at 4 °C (Duppont-RC5). The exopolymer was precipitated with ethanol (−80 °C at three times the volume). The pellet was centrifuged at 13,154× *g* for 30 min at 4 °C and dialyzed with Milli-Q water for 48 h. For further purification, chromatography of the exopolymer (10 mL, 10 mg/mL) in a DEAE-52 anion exchange column (2.6 × 30 cm) eluted with deionized water, 0.05 and 0.3 M NaCl, at a flow rate of 1 mL/min rate was performed.

### 3.3. MTT Assay

The cytotoxicity activity was determined by reducing the MTT (3-[4,5-dimethyl-thiazol-2-yl]-2,5-diphenyltetrazolium bromide) to formazan on HeLa cells [35,36]. The HeLa were cultured on 24-well plates and reached a density of 5 × 10^5^ cells/per well and treated with different concentrations of EPSp (200, 400, 600, 800, and 1000 µg/mL) for 24 h. Salt solution (0.5 mg/mL) was added and incubated for 4 h under 5% CO_2_ atmosphere incubator and at 37 °C. Subsequently, 100 μL of SDS (10%) in 0.01 M of HCL was added to each well to dissolve the formazan crystals for 30 min. The absorbance was measured at 490 nm, and, finally, the cell viability was calculated.

(1) Cell viability [%]=(A1/A2)×100

A1 = cells treated with EPSp and MTT salt solution, and A2 = cells without any treatment with MTT salt solution.

### 3.4. Free Radical Scavenging Activities

#### 3.4.1. DPPH (1,1-Diphenyl-2-picryl Hydrazyl Radical) Radical Scavenging Activity

The DPPH radical scavenging effect of the EPSp was measured using the method described in [8,37]. The sample solution was prepared in a final volume of 150 μL. This contained 50 μL of EPSp at different concentrations (0.1, 0.25, 0.8, 1.0, 2.5, 5.0, 7.5, and 10 mg/mL) and 100 μL DPPH (100 μM DPPH–ethanolic solution). The control solution was prepared by adding 50 μL Milli-Q water with 100 μL DPPH–ethanol solution. The positive control was vitamin C (Vc). Then, the mixtures were incubated in the dark for 30 min at room temperature. The absorbance of the DPPH radicals was determined at 525 nm. Percentage DPPH radical scavenging activity was calculated by the equation:

(2) DPPH scavenging activity [%]=[1−(A sample−A control)/A blank]×100

(3) A blank=50 μL Milli Q water+100 μL de ethanol 96%

(4) A control=50 μL Milli Q water+100 μL de DPPH−ethanol solution

(5) A sample=50 μL (EPS or Vc)+100 μL de DPPH−ethanol solution

#### 3.4.2. Hydroxyl Radical (OH) Scavenging Activity

The hydroxyl radical scavenging activity effect of the EPSp was measured as described previously [8,38]. The sample solutions contained 40 μL of EPSp at different concentrations (0.1, 0.25, 0.8, 1.0, 2.5, 5.0, 7.5, and 10 mg/mL), 40 μL of salicylic acid (9 mM ethanol–salicylic acid solution), 40 μL FeSO_4_ solution (9 mM), and 40 μL of H_2_O_2_ (8.8 mM). The positive control was vitamin C (Vc). The mixtures were incubated for 30 min at 37 °C. The absorbance of the hydroxyl radical was determined at 510 nm. Percentage hydroxyl radical scavenging activity was calculated by the equation:

(6) OH scavenging activity [%]=[1−(A sample−A control)/A blank]×100

(7) $$A\;blank=40\;\mu L\;Milli\;Q\;water+40\;\mu L\;{FeSO}_{4}\;solution+40\;\mu L\;de\;ethanol\;96\%+40\;\mu L\;of\;H_{2}O_{2}$$

(8) $$A\;control=40\;\mu L\;Milli\;Q\;water+40\;\mu L\;{FeSO}_{4}\;solution+40\;\mu L\;de\;ethanol-salicylic\;solution+40\;\mu L\;of\;H_{2}O_{2}$$

(9) $$A\;sample=40\;\mu L\;(EPS\;or\;Vc)+40\;\mu L\;{FeSO}_{4}\;solution+40\;\mu L\;de\;ethanol-salicylic\;solution+40\;\mu L\;of\;H_{2}O_{2}$$

#### 3.4.3. Superoxide Anion (O_2−_) Radical Scavenging Activity

The superoxide anion radical scavenging activity effect of the EPSp was measured as previously described [8,39]. The sample solutions contained 0.3 mL of EPSp at different concentrations (0.1, 0.25, 0.8, 1.0, 2.5, 5.0, 7.5, and 10 mg/mL), Then, 2.6 mL of phosphate buffer (50 mM, pH 8.2) and 90 μL of pyrogallol (3 mM) and HCl (10 mM) were added to the sample solutions. The positive control was vitamin C (Vc). The absorbance of superoxide anion radicals was determined at 325 nm. Percentage of the superoxide anion radical scavenging was calculated by the equation:

(10) $$O_{2-}\;scavenging\;activity\;[\%]=[1-(A\;sample\;10/A\;control\;10)-(A\;sample\;0/A\;control\;0)]\times 100$$

(11) A control (0 min and 10 min)=0.3 mL Milli Q water+2.6 mL fosfate Buffer+90 mL pyrogallol−HCl

(12) A sample (0 min and 10 min)=0.3 mL (EPS or Vc)+2.6 mL fosfate Buffer+90 mL pyrogallol−HCl

### 3.5. H_2_O_2_-Induced Assay

#### 3.5.1. H_2_O_2_-Induced Oxidative Stress

H_2_O_2_-induced oxidative stress was used to measure the damage of this non-radical on HeLa cells [8,40,41]. For the in vitro procedure, the cells were seeded in 96-well plates 5 × 10^4^ for 24 h, under 5% CO_2_ atmosphere incubator, and at 37 °C. Subsequently, the medium was replaced with 100 μL of different concentrations of H_2_O_2_ (0.25, 0.5, 1, and 2 mM) for 1 h at 37 °C. After this time, the medium was removed, and cell viability estimated by MTT method as described in Section 3.3.

#### 3.5.2. Effects of the Exopolymer against H_2_O_2_

The effect of the EPSp against the non-radical H_2_O_2_ was evaluated on HeLa cells [8,40,41]. The cells were seeded in 96-well plates of 5 × 10^4^ for 24 h, under 5% CO_2_ atmosphere incubator and at 37 °C. After this time, the EPSp was diluted in a new medium with DMEM at different concentrations (25, 50, 100, 200, and 400 μg/mL) for 1 h. The positive control was vitamin C (Vc) (20 mg/mL). Cell viability was estimated by MTT method as described in Section 3.3.

### 3.6. Enzymatic Antioxidant Assays

#### Catalase (CAT) and Glutathione Peroxidase (GSH-Px) Assay

EPSp was investigated against oxidative stress on HeLa cells [41,42]. For it, 5 × 10^5^ cells were seeded in 24-well plates and incubated for 24 h. Cells were treated with EPS at various concentrations (50, 100, 200, and 400 μg/mL) for 1 h at 37 °C. The supernatants were collected and used for the determination of CAT and GSH-Px assay. The determination of the reactions was carried out using the kit (Sigma) established in the protocol.

### 3.7. Emulsifying Properties

The emulsifying properties of EPSp were evaluated using the method described by Meneghine et al. [43]. The assays were undertaken in transparent cylindrical 5 mL tubes that contained 1.5 mL of an oil phase and 1.5 mL of an aqueous phase, at two different times (24, and 168 h), in different concentrations (0.5, 1, and 2 mg/mL), and at pH 7.0. The oil phase contained vegetable oils (sunflower oil, olive oil, sesame oil, and coconut oil). For the aqueous phase, both commercial emulsifying compounds, such as polysorbate 20 (Tween 20) (Sigma), sodium dodecyl sulphate (SDS) (Sigma), and Triton X-100 (Sigma), and the obtained polymers were used for comparison purposes. All compounds used had a concentration of 3:2 *v*/*v*. The tubes were stirred in a vortex at 2400 rpm for 2 min. After 24 h and 168 h, the emulsification indices E24 and E168 were determined by the equation:

(13) E [%]=HEL/HT×100

where HEL (mm) is the height of the emulsion layer and HT (mm) is the overall height.

### 3.8. Statistical Analysis

All experiments were carried out in triplicate. The data were recorded as mean ± standard deviation and analyzed by SPSS (version 21, SPSS^®^ Inc., Chicago, IL, USA). One-way analysis of variance was performed by ANOVA procedures. Significant differences between means were determined by Duncan’s multiple-range tests. *p* < 0.05 was considered statistically significant.

## 4. Conclusions

The EPSp exhibited the absence of cytotoxicity and presented strong hydroxyl radical (OH), DPPH scavenging, and superoxide anion (O_2−_) activities. In addition, EPSp had significant antioxidant activity against H_2_O_2_-induced injury, protecting HeLa cells from oxidative stress. EPSp also stimulated the activities of the antioxidant enzymes Catalase (CAT) and Glutathione peroxidase (GSH-Px). The performance of EPSp as an emulsifier is notable in the case of sesame oil, as it had a higher performance than commercial emulsifiers. This study suggests that the EPSp negative charges of *Bacillus licheniformis* played an important role in shaping potential applications. EPSp’s performance in the evaluated assays suggests its versatility and suitability for various biotechnological applications.

The exploration of a broad spectrum of biotechnological activities of the EPS obtained by *Bacillus licheniformis* demonstrated excellent antioxidant activity, both non-enzymatic and enzymatic, protection against H_2_O_2_-induced oxidative stress, emulsifying properties, and, previously, antiviral capabilities. This type of deeper study allows us to have a more realistic vision of the application of exopolysaccharides produced by micro-organisms, and their competitive advantages at an industrial level.

## Data Availability

No new data were created or analyzed in this study. Data sharing is not applicable to this article.
